# A Case Report on the Detection of Anti-M Antibodies in a Pediatric Patient With Pneumonia

**DOI:** 10.7759/cureus.87440

**Published:** 2025-07-07

**Authors:** Digeet Davad, Param H Salot, Harsh Majithiya, Jay Nagda

**Affiliations:** 1 Department of Immunohematology and Blood Transfusion, Shri M P Shah Government Medical College, Jamnagar, IND; 2 Department of Pathology, Shri M P Shah Government Medical College, Jamnagar, IND; 3 Department of Community Medicine, Shri M P Shah Government Medical College, Jamnagar, IND

**Keywords:** alloimmunization, anti-m antibody, pediatric transfusion, pneumonia, serological testing

## Abstract

Blood group antibodies can pose serious challenges during transfusion, especially in pediatric patients, where immune responses may be unpredictable. Early identification of alloantibodies helps guide safe transfusion practices. We present the case of an 18-month-old female patient diagnosed with pneumonia, in whom an anti-M antibody was detected during pre-transfusion testing. The patient, admitted to Guru Gobind Singh Government Hospital in Jamnagar, India, in January 2025, required a blood transfusion due to severe anemia (hemoglobin 7.1 g/dL). Serological testing revealed a positive antibody screen with anti-M antibody identified via three-cell and 11-cell panels. This case highlights the importance of advanced serological testing in pediatric patients requiring transfusion, particularly those with underlying infections like pneumonia, which may complicate transfusion management.

## Introduction

The MNS blood group system, which includes the M antigen, plays a critical role in transfusion medicine due to its ability to stimulate alloimmune responses. Anti-M antibodies, although relatively uncommon, are important to identify during pre-transfusion testing as they can complicate transfusion decisions [1]. These antibodies may be naturally occurring and typically belong to the Immunoglobulin-M (IgM) class, but in some cases, they may also be Immunoglobulin-G (IgG), which are clinically significant and may cause hemolytic transfusion reactions [2].

An alloantibody refers to an immune response directed against red cell antigens not present on the patient’s own red cells, commonly occurring following blood transfusion or pregnancy. The indirect antiglobulin test (IAT) is used to detect these antibodies in patient plasma prior to transfusion.

In pediatric patients, infections such as pneumonia often lead to systemic inflammation and bone marrow suppression, resulting in anemia that necessitates blood transfusion [3]. When alloantibodies like anti-M are present, transfusion management becomes more complex. This report presents the case of an 18-month-old female with pneumonia and anemia, in whom a clinically significant anti-M antibody was detected, highlighting the role of pre-transfusion antibody screening in pediatric care.

## Case presentation

**Figure 1:**
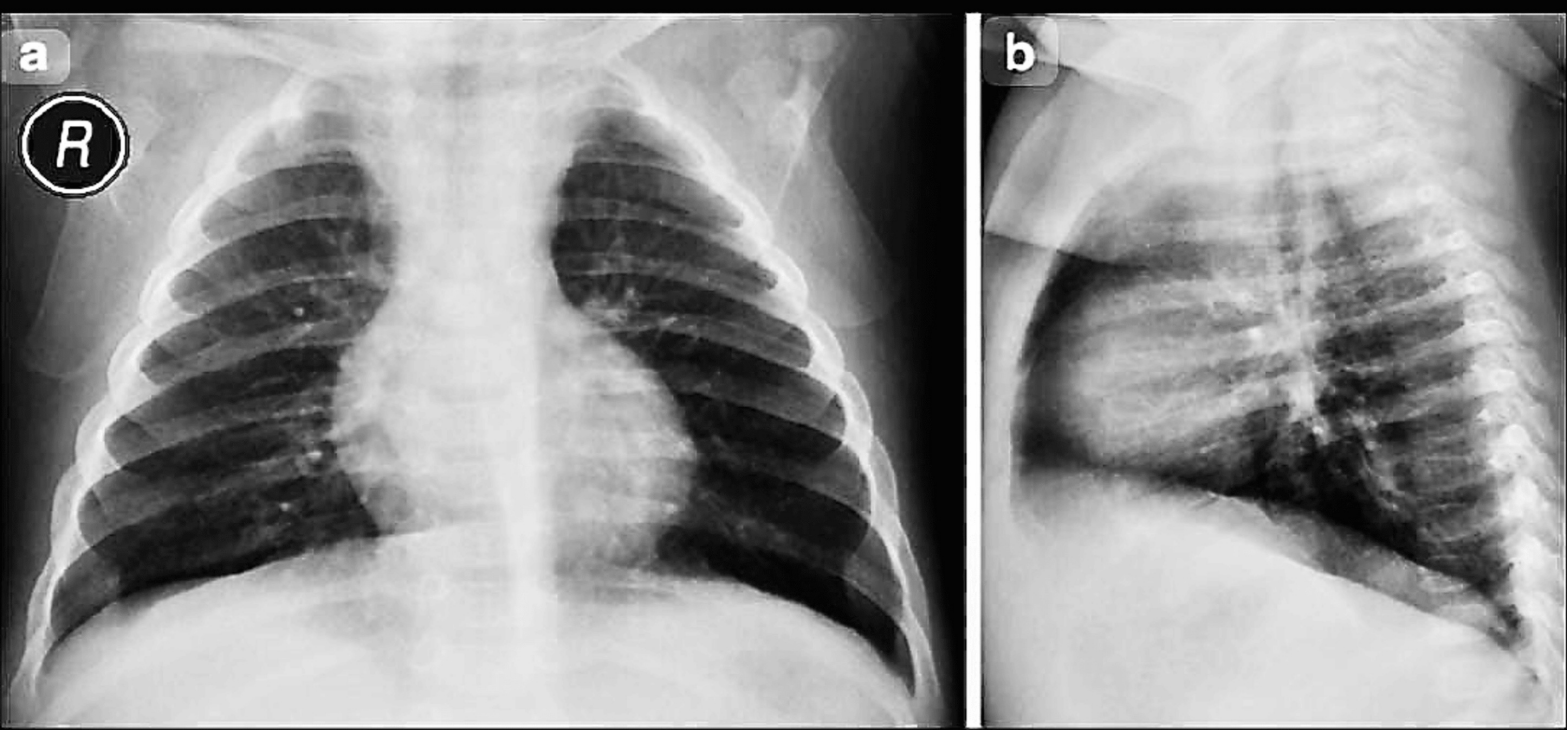
Posteroanterior and lateral chest radiographs of the pediatric patient Chest radiograph of an 18-month-old female presenting with pneumonia. (a) Posteroanterior view showing bilateral pulmonary opacities, predominantly involving the right middle and lower zones, consistent with alveolar consolidation. The cardiac silhouette appears normal for age, and no pleural effusion or pneumothorax is seen. (b) Lateral view further confirms consolidation in the posterior segments of the right lower lobe, supporting the diagnosis of lobar pneumonia.

An 18-month-old female presented to Guru Gobind Singh Government Hospital, Jamnagar, in January 2025, with a history of fever, cough, and respiratory distress. Clinical evaluation confirmed a diagnosis of pneumonia, supported by chest imaging (Figure 1). Her vital signs included a blood pressure of 90/50 mmHg and a mean arterial pressure of 64 mmHg. 

On admission, the child presented with severe anemia and signs of systemic inflammation. Hemoglobin was 7.1 g/dL (reference: 11-14 g/dL), with a low MCV of 50 fL (normal: 70-86 fL) and reticulocyte count of 0.4% (normal: 0.5%-1.5%), indicating a hypo-regenerative anemia pattern, likely secondary to marrow suppression due to infection. Leukocytosis (15200/mm³; ref: 4000-11000/mm³) and thrombocytosis (4.8 lakh/mm³; ref: 1.5-4 lakh/mm³) were consistent with acute systemic inflammation. The positive IAT and antibody screening confirm the presence of a significant anti-M alloantibody (Table 1).

**Table 1:** Laboratory investigations of the patient on admission This table summarizes the hematological and immunohematological findings of an 18-month-old female patient diagnosed with pneumonia and anemia. Laboratory values include hemoglobin, red cell indices, leukocyte and platelet counts, as well as results from serological testing. Elevated WBC and platelet counts are indicative of an ongoing infectious process, while the low hemoglobin and reticulocyte counts suggest hypo-regenerative anemia. DAT: direct antiglobulin test; IAT: indirect antiglobulin test

Parameter	Patient value	Normal reference range
Hemoglobin	7.1 g/dL	11.0-14.0 g/dL (children, 6 months-6 years)
MCV	50 fL	70-86 fL
Reticulocyte count	0.4%	0.5%-1.5%
Total leukocyte count (WBC)	15200 /mm³	4000-11000 /mm³
Platelet count	480000 /mm³	150000-400000 /mm³
DAT	Negative	Negative
IAT	Positive (2+)	Negative
Antibody screening	Positive (anti-M antibody)	Negative

**Figure 2:**
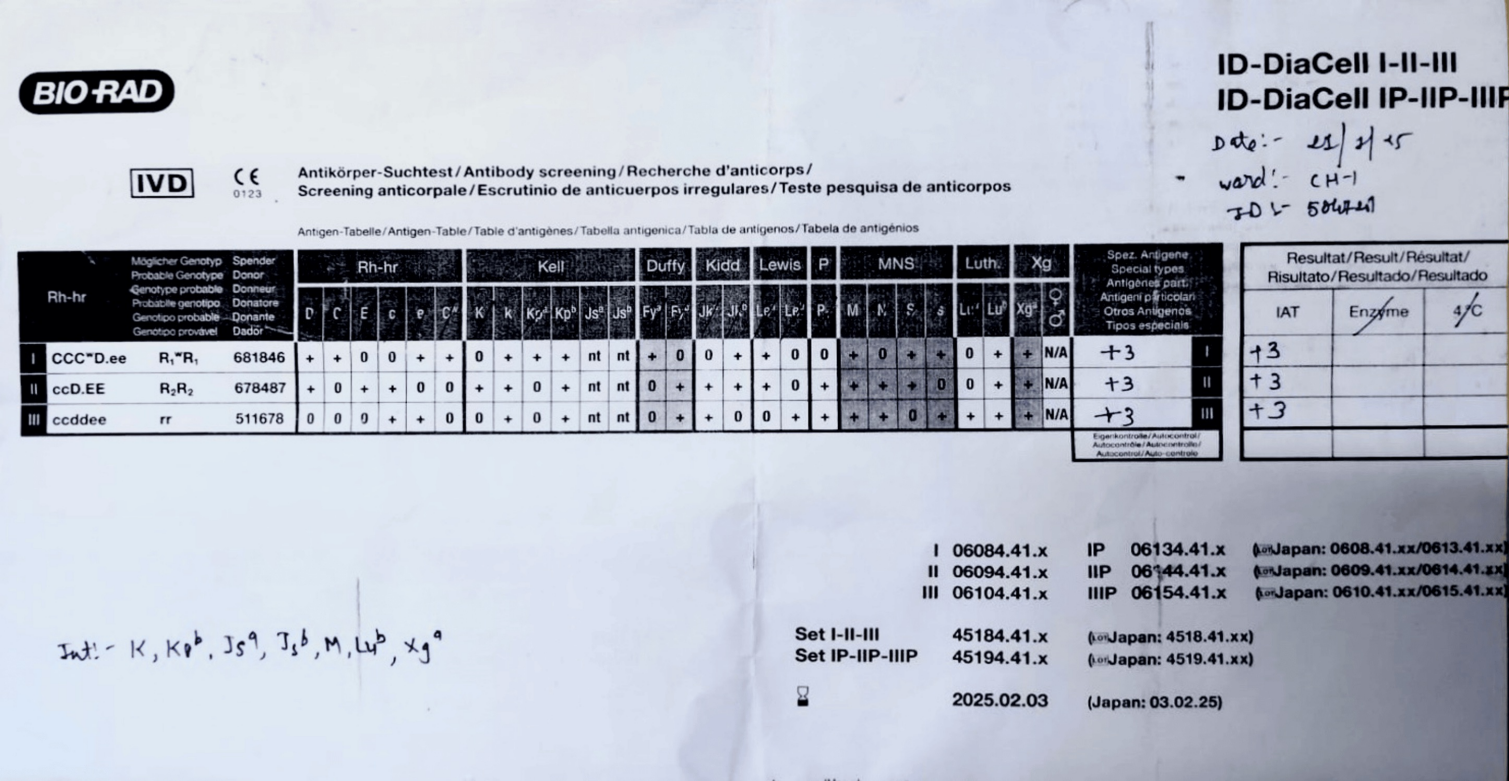
Antibody screening using a three-cell panel by the column agglutination method Antibody screening results using the Bio-Rad ID-DiaCell I-II-III panel showed strong pan-reactivity (3+, 3+, 3+) with all three reagent cells. The IAT was positive (enzyme phase not reactive), indicating the presence of a clinically significant alloantibody. The pattern of reactivity corresponds to anti-M specificity, as later confirmed through extended panel testing. This serological profile supports the identification of an alloantibody that requires antigen-negative red cell units for transfusion. IAT: indirect antiglobulin test

Due to her anemia and clinical status, a blood transfusion was requested on January 20, 2025. Pre-transfusion testing was performed at the Department of Immunohematology and Blood Transfusion, Guru Gobind Singh Government Hospital, Jamnagar. The direct antiglobulin test (DAT) was negative, ruling out autoimmune hemolytic anemia, but the IAT was positive (Grade 2+). Antibody screening using a three-cell panel (by column agglutination method) showed strong positivity (+3) across all cells (Figure 2). Further testing with an 11-cell panel confirmed the presence of anti-M antibody, with a reaction pattern of +3 in cells 1, 4, 5, 7, 9, 10, and 11, and no reaction in cells 2, 3, 6, and 8, as well as a negative auto-control (Table 2).

**Table 2:** Antibody screening with an 11-cell panel (by the column agglutination method) Antibody screening results using an 11-cell panel. The reaction pattern demonstrates strong positivity (+3) with cells 1, 4, 5, 7, 9, 10, and 11, and no reactivity (0) with cells 2, 3, 6, and 8. The auto-control is negative, indicating the presence of an alloantibody rather than an autoantibody. This reactivity pattern is consistent with anti-M antibody specificity.

Panel cell	Cell 1	Cell 2	Cell 3	Cell 4	Cell 5	Cell 6	Cell 7	Cell 8	Cell 9	Cell 10	Cell 11	Auto-control
Grade	+3	0	0	+3	+3	0	+3	0	+3	+3	+3	0

The patient’s blood group is O+ve, and her clinical diagnosis of anemia secondary to pneumonia necessitated the selection of M-antigen negative red blood cell units for transfusion. One unit of packed red blood cells (PRBCs) was requested for routine transfusion on January 20, 2025, at 7:20 PM. Post-transfusion, the patient’s hemoglobin improved, and no adverse transfusion reactions were reported. She was discharged on January 24, 2025, after clinical stabilization, with a follow-up plan for monitoring her hemoglobin levels.

## Discussion

Anti-M antibodies are relatively uncommon but can pose significant challenges in transfusion, particularly in pediatric patients, where the immune response may be less predictable [4]. In this case, the anti-M antibody was identified in an 18-month-old child with pneumonia, a condition known to cause anemia due to systemic inflammation and bone marrow suppression [3]. Naturally occurring anti‑M has also been reported in infants with acute infections such as pyelonephritis [4]. The negative DAT and positive IAT in our case suggested an alloantibody rather than an autoimmune process, which was confirmed by the 11-cell panel identifying anti-M specificity.

The 11-cell antibody panel showed strong reactivity (+3) with cells 1, 4, 5, 7, 9, 10, and 11, while cells 2, 3, 6, and 8 showed no reaction. The auto-control was negative, confirming that the detected antibody was an alloantibody, not an autoantibody. This serological pattern matched anti-M specificity, guiding the transfusion team to select M antigen-negative red blood cell units to ensure compatibility.

The clinical significance of anti-M antibodies varies: while they are often IgM and cold-reactive, IgG anti-M can cause hemolytic transfusion reactions [2]. Although usually cold-reactive and benign, IgM anti‑M has caused acute hemolytic transfusion reactions [5]. Rare cases of biphasic anti‑M antibodies with both IgM and IgG activity have caused hemolytic disease of the fetus and newborn (HDFN) [6]. In severe alloimmunization, anti‑M has necessitated intra‑uterine transfusions to prevent fetal anemia [7]. Anti‑M antibodies may pose risks even during pregnancy, as highlighted in recent literature [8].

In our patient, the use of M antigen-negative units mitigated this risk, highlighting the importance of advanced serology in transfusion medicine. Detection of anti‑M antibodies has also been shown to delay transfusion services and increase costs in hospital settings [2]. Following transfusion with M antigen-negative PRBCs, the patient’s hemoglobin rose to 10.2 g/dL, and no signs of transfusion reaction were observed. This favorable outcome confirmed the effectiveness of targeted antigen-matching based on serological findings. The patient’s young age and underlying infection may have contributed to her immune response, though the exact mechanism of anti-M development remains unclear. Although prior transfusion history was not documented, the absence of any previous reaction and the young age of the patient suggest the anti-M antibody may have been naturally occurring or unmasked by the inflammatory state caused by pneumonia.

This case underscores the need for comprehensive pre-transfusion testing in pediatric patients with acute infections, as alloantibodies can complicate management. A prospective donor study found anti‑M in 1.4% of healthy individuals, supporting its role in routine screening [9]. Anti‑M is among the commonly identified alloantibodies in pediatric transfusion recipients, complicating compatibility matching [10].

This is a single case report, and while it highlights an important transfusion concern, its findings cannot be generalized. Further prospective studies are needed to assess the prevalence, immunogenic triggers, and clinical significance of anti-M antibodies in pediatric populations with acute infections.

## Conclusions

The detection of an anti-M antibody in an 18-month-old female with pneumonia highlights the critical role of comprehensive serological testing in ensuring safe transfusion practices in pediatric patients. This case demonstrates the successful management of anemia in a child with an alloantibody, using antigen-negative red cell units, without any adverse transfusion reactions. The patient’s hemoglobin improved post-transfusion to 10.2 g/dL, and she was discharged in stable condition, reinforcing the importance of individualized transfusion strategies based on advanced immunohematological evaluation. While this is a single case report and its generalizability is limited, it underscores the need for vigilance among clinicians regarding unexpected antibodies in children with infections, especially when anemia requires transfusion. Future studies are warranted to better understand the prevalence, origin, and clinical implications of anti-M antibodies in pediatric populations.
